# Exploring the impact of mass migration in Jin Dynasty by psycholinguistic analysis on Classical Chinese legacy text

**DOI:** 10.3389/fpsyg.2022.993141

**Published:** 2022-11-15

**Authors:** Huan Xiong, Gengxuan Wu, Sijia Li, Shujie Xue, Hua Li, Tingshao Zhu

**Affiliations:** ^1^Institute of Psychology, Chinese Academy of Sciences, Beijing, China; ^2^Department of Psychology, University of Chinese Academy of Sciences, Beijing, China; ^3^Institute of Qilu Culture, Shandong Normal University, Jinan, China

**Keywords:** psycholinguistic analysis, word frequency analysis, LIWC, Classical Chinese legacy text, mass migration, gentry of the Jin Dynasty

## Abstract

The first mass migration in China took place at the end of the Western Jin, which resulted in the southward transfer of the Central Plains Culture and brought about huge social changes. Such social changes exerted significant impacts on the gentry of the Jin Dynasty. This paper used a huge volume of Classical Chinese legacy text of Jin gentry members. We used CC-LIWC to calculate frequencies of different word categories used in these text contents and conducted an analysis of variance to measure significant differences between the three groups. We found 16 categories of words with significant differences and calculated their effect sizes, such as tense markers (tensem), *F* = 3.588, *P* < 0.05, η^2^ = 0.034; modal particles (modal_pa), *F* = 3.468, *P* < 0.05, η^2^ = 0.053; words for affective processes (affect), *F* = 3.096, *P* < 0.05, η^2^ = 0.028; words for cognitive processes (cogproc), *F* = 3.308, *P* < 0.05, η^2^ = 0.031; words for perceptual processes (percept), *F* = 7.137, *P* < 0.05, η^2^ = 0.06. Combining the psycholinguistics of the 16 categories of words and researches of historians on the Jin Dynasty, we then analyzed the direct and indirect, immediate and long-lasting psycholinguistic impacts of this mass migration on the gentry themselves and their descendants.

## Introduction

Historical events inevitably had impacts on people, which could be expressed and recorded in legacy text (Zhang, 2021). There were three mass migrations in China, which led to the transfer of the Central Plains Culture to the south (Chen, 2016). The first mass migration took place during 311–317 A.D., in which the Western Jin came to an end and the Eastern Jin was established since then. The loss of control over the north and being cornered to the south cast huge shadows on the mentality of the gentry.

Researchers have compared the writings by Western and Eastern Jin gentry, mainly focusing on spiritual perplexities, value losses, and emotional depressions (Wang, 2021) caused by the mass migration. The Jin gentry was found to have a conflicting philosophy of life that embodies both romantic regimen and heavy-hearted sentiments (Zhou, 2001). Literature and art of the Western Jin mainly manifested secular spirits while those of Eastern Jin embodied transcendent spirits (Chen, 2017). Previous researches mainly adopted speculative or qualitative methods, in which only a small number of writings were analyzed to identify the psychological impacts on the Jin gentry (Fu, 2005); moreover, previous researches mainly analyzed such changes from the perspectives of political changes (Luo, 2005), philosophical trends (Luo, 2005) and artistic spirits (Chen, 2017), but not the gentry members themselves.

It might be the mass migration that contributed to how the Eastern Jin differed from the Western Jin. Yuqing Tian summarized Eastern Jin politics as “clan-dominated politics” featuring “a balance between idle emperors, powerful clans, and exploited displaced people (Tian, 1996).” Due to the mass migration, different factions of gentry clans took shape: gentry clans emigrated from the north and aboriginal gentry clans of the south, and the latter was subordinate to the former during the Eastern Jin (Yan, 1996).

The impacts on the gentry caused by the mass migration were also reflected in the ideological and cultural trends. Ideologically, on the one hand, the southward gentry were ashamed of losing the north (Wang, 2016); on the other hand, the gentry manifested a sense of responsibility and expressed the intent to do something to change the situation. The psychological changes caused by the mass migration released more literature potential, contributing to the cultural prosperity during the Eastern Jin.

In the recent past, cultural product analysis has been increasingly preferred in examining cultural changes, and an archive of written language is suitable for cultural product analysis (Hamamura, 2015). Zeng and Greenfield used the Google Ngram Viewer to calculate changing frequencies of certain key words to study the implications of social and political changes on cultural values in China over the last 40 years (Zeng and Greenfield, 2015). Duran and Gruber uses LIWC (Linguistic Inquiry and Word Count) to study the deception of language (Duran et al., 2010), and the implicit achievement motivation of words (Gruber, 2022). Hu et al. (2021) used CC-LIWC (LIWC for Classical Chinese) to calculate the frequencies of “Ren” and “Li” in SikuQuanshu to investigate the changes of Confucian culture across 2000 years.

This paper tried to analyze the impacts of the mass migration on the gentry by psycholinguistic analysis. This work extended the research scope as previous studies focused on changes to geography (Tang, 2014), literature (Ma, 2019) and arts (Chen, 2017) than on changes to people’s psychology. As a Classical Chinese psycholinguistic program, CC-LIWC can output the psycholinguistic word frequency values of massive texts through language exploration and word counting, In this study, we used CC-LIWC to conduct psycholinguistic analysis, which has been used to analysis Su Shi’s personality and emotions changed after the Crow Terrace Poetry Trial (Ma, 2020) and the psycholinguistic changes of Zhang Juzheng after he chose to remain in office instead of entering filial mourning for his father’s death (Li, 2020). Using CC-LIWC, we analyze the psycholinguistic changes of a group of people before, during and after the mass migration.

“Northern clans fled to the south to escape the warfare” (from a poem by Du Fu). To study the psycholinguistic effects of this life-changing event on a group of people, this paper used a psycholinguistics-based quantitative tool to analyze the legacy text of these people that were divided into three groups based on the timeline.

## Materials and methods

The object of this research is self-expressive legacy text written by the gentry members of the Jin Dynasty. After checking many historical documents on the Jin Dynasty, we finally selected *Quan Jin Wen* (Yan, 1999) as the major source for analysis. *Quan Jin Wen* is a collection of writings by Jin gentry members, including their letters, diaries, essays, etc. Based on the dates of birth and death as well as working periods of the Jin gentry members, we divided their legacy text into three groups.

The pre-migration group includes 102 gentry members, with 1,096 articles and totaling 287,093 Chinese characters. Text falling into this group meet the following criteria: written by gentry members who born during or before the Western Jin Dynasty (265–316); died during the Western Jin; born, worked and lived in the north.

The migration group includes 57 gentry members, with 525 articles and totaling 102,034 Chinese characters. Text falling into this group meet the following criteria: written by gentry members who born during or before the Western Jin Dynasty (265–316); died during the Eastern Jin; originally worked and lived in the north and then in the south after the southward migration; peer, children or grandchildren of the Western Jin gentry. Writers of this group are collectively called the “southward gentry” hereafter.

The post-migration group includes 57 gentry members, with 565 articles and totaling 126,951 Chinese characters. Text falling into this group meet the following criteria: written by gentry members who born during the Eastern Jin Dynasty (317–420); died during the Eastern Jin; born, worked and lived in the south; friends or children or grandchildren of the southward gentry.

After more than 20 years of development, LIWC (Pennebaker, 2011) has been widely used by researchers with satisfactory reliability and validity (Tausczik and Pennebaker, 2010), and it can be used in multiple languages including Chinese (Zhang, 2015). The data processing tool used by this paper is the Classical Chinese LIWC (CC-LIWC) (Fan, 2020), a dictionary program developed by the Computational Network Psychology Laboratory at the Institute of Psychology, Chinese Academy of Sciences based on the simplified Chinese version of the LIWC dictionary (SC-LIWC) (Fan, 2020). With this program, we calculated the frequencies of 80 psycholinguistic LIWC categories (Fan, 2020) used in the above three groups.

Note that each sample in the three groups is a collection of all articles from one writer (gentry member), and different samples correspond to the articles of different writers. We then applied the SPSS Statistics 26 to analyze the CC-LIWC-produced word frequency data of the three groups, conducted a one-way analysis of variance (ANOVA) based on the only variable of time (in this case, before, during and after the mass migration), and finally used the Fisher’s Least Significant Difference (LSD-*F*) method to run a *post-hoc* test of the results with significant differences, calculate their effect sizes and other relevant indicators.

ANOVA is used to test the significance of the difference between the mean values of two or more samples. ANOVA can only judge whether there is a difference between the overall mean values. A *post-hoc* test can be used to further determine which two variables have differences and which two have not. LSD-F is a common *post-hoc* test method. In the calculation of standard error, it fully considers the sample information at all levels of the population, and estimates a more robust standard error. To avoid increasing the probability of making a type I error (i.e., false positive) when performing multiple hypothesis testing, we use the FDR method for correction. Due to the sample size, it is also necessary to calculate the effect size. The main indexes and formulas involved are as follows:


$$F=M\,S_{b}/M\,S_{w}$$

(*MS*_*b*_ is the mean square between groups. *MS*_*w*_ is the mean square within group.)


P=p⁢i*(n/r)

(*pi* is the *p* value calculated by *LSD-F*. *n* is the number of *p* values. *r* is *n, n-1,…,1*.)


$$\eta^{2}=S\,S_{b}/S\,S_{t}$$

(*SS*_*b*_ means between groups sum of squares. *SS*_*t*_ means total sum of squares.)

## Results

After calculating the frequencies of 80 psycholinguistic LIWC categories used in the three groups of text with the CC-LIWC, we then ran ANOVA (Rutherfoord, 2001) and a *post-hoc* test, from which we found 16 categories of words with significant differences. Their *p*-values, *f*-values and effect sizes (η^2^) are as follows:

*auxiliary verbs (auxverb)*, *F* = 3.899, *P* < 0.05, η^2^ = 0.032;

*negative words (negate)*, *F* = 6.415, *P* < 0.05, η^2^ = 0.047;

*postpositions (postpend)*, *F* = 4.032, *P* < 0.05, η^2^ = 0.071;

*tense markers (tensem)*, *F* = 3.588, *P* < 0.05, η^2^ = 0.034;

*modal particles (modal_pa)*, *F* = 3.468, *P* < 0.05, η^2^ = 0.053;

*interrogative words (interrog)*, *F* = 3.34, *P* < 0.05, η^2^ = 0.0;

*quantifiers (quant)*, *F* = 3.561, *P* < 0.05, η^2^ = 0.037;

*words for affective processes (affect)*, *F* = 3.096, *P* < 0.05, η^2^ = 0.028;

*words for sadness (sad)*, *F* = 4.588, *P* < 0.05, η^2^ = 0.04;

*words for cognitive processes (cogproc)*, *F* = 3.308, *P* < 0.05, η^2^ = 0.031;

*words for insight (insight)*, *F* = 4.86, *P* < 0.05, η^2^ = 0.042;

*tentative words (tentat)*, *F* = 4.027, *P* < 0.05, η^2^ = 0.032;

*words for perceptual processes (percept)*, *F* = 7.137, *P* < 0.05, η2 = 0.061;

*words for seeing (see)*, *F* = 5.944, *P* < 0.05, η^2^ = 0.05;

*words for feelings (feel)*, *F* = 6.351, *P* < 0.05, η^2^ = 0.05;

*words for leisure (leisure)*, *F* = 4.435, *P* < 0.05, η^2^ = 0.035.

In particular, these p values were FDR corrected values, which effectively controlled the false positive rate (Benjamini and Hochberg, 1995). Although η^2^ were not large, however according to J. Cohen’s criteria for determining the effect of variance analysis of η^2^, it is a small effective size when η^2^ = 0.01, a medium effective size when η^2^ = 0.06, and a large effective size when η^2^ = 0.14 (Cohen, 1988). Some of the η^2^ values in this paper were medium effective size which was available.

We focused on the *post-hoc* test results of negative words, tense markers and modal particles for detailed discussion. For more test results, Table 1 shows the differences in the frequencies of different categories of words used in the three groups and Table 2 presents the correlation of relevant indicators of the 16 categories of words.

**TABLE 1:** Differences in the frequencies of different categories of words used in the three groups.

Category	Word examples	M ± SD			*F*	*P*	η^2^
					
		Pre-migration (*n* = 102)	Migration (*n* = 57)	Post-migration (*n* = 57)			
auxverb^c^	may, must, can	0.0133 ± 0.0096	0.0178 ± 0.0131	0.0179 ± 0.0139	3.899	0.035*	0.032
negate^ab^	no, not, none	0.0315 ± 0.0124	0.0396 ± 0.0158	0.0341 ± 0.014	6.415	0.011*	0.047
postpend	up, down	0.0143 ± 0.0081	0.0127 ± 0.0085	0.0105 ± 0.0074	4.032	0.034*	0.071
tensem^ab^	now, current, future	0.0301 ± 0.0112	0.0341 ± 0.0127	0.0286 ± 0.0108	3.588	0.040*	0.034
modal_pa^ab^	alas, ackaday, alack	0.0137 ± 0.0105	0.0099 ± 0.007	0.0137 ± 0.0092	3.468	0.041*	0.053
interrog^c^	why, how, who	0.0077 ± 0.0076	0.0069 ± 0.0049	0.01 ± 0.007	3.34	0.041*	0.000
quant^c^	one, many, only	0.0272 ± 0.0108	0.0246 ± 0.0097	0.0302 ± 0.013	3.561	0.040*	0.037
affect^c^	emotion, feeling	0.1562 ± 0.0294	0.1596 ± 0.0268	0.1679 ± 0.0284	3.096	0.047*	0.028
sad^c^	loss, death	0.0173 ± 0.0095	0.0201 ± 0.017	0.0248 ± 0.0199	4.588	0.029*	0.040
cogproc^b^	believe, so, know	0.145 ± 0.0304	0.1427 ± 0.0338	0.1585 ± 0.0475	3.308	0.041*	0.031
insight^b^	thought, understood	0.0457 ± 0.0195	0.0472 ± 0.0164	0.0561 ± 0.0258	4.86	0.029*	0.042
tentat^b^	if, or	0.0251 ± 0.0111	0.0255 ± 0.0103	0.0305 ± 0.0145	4.027	0.034*	0.032
percept^b^	say, speak, reckon	0.06 ± 0.0218	0.0568 ± 0.0196	0.0716 ± 0.0257	7.137	0.011*	0.061
see^b^	see, look, observe	0.0207 ± 0.0132	0.0204 ± 0.0106	0.0277 ± 0.0156	5.944	0.012*	0.050
feel^b^	heavy, light, loose	0.0117 ± 0.0076	0.0107 ± 0.0077	0.0165 ± 0.0133	6.351	0.011*	0.050
leisure^b^	travel, drink, taste	0.0145 ± 0.013	0.0134 ± 0.011	0.0214 ± 0.0235	4.435	0.030*	0.035

M is the mean, SD is the standard deviation, *P* is the *P* value corrected by FDR (keep three decimal places).

^a^There are differences between the migration group and the pre-migration group. ^b^There are differences between the migration group and the post-migration group.

^c^There are differences between the pre-migration group and the post-migration group.

*P < 0.05.

**TABLE 2:** Correlation matrix of the 16 categories of words.

	auxverb	negate	prepend	tensem	modal_pa	interrog	quant	affect	sad	cogproc	insight	tentat	percept	see	feel	leisure
auxverb																
negate	0.356**															
prepend	−0.173*	−0.150*														
tensem	–0.067	0.062	0.165*													
modal_pa	–0.092	–0.009	–0.081	–0.054												
interrog	−0.140*	0.002	–0.096	–0.04	0.423**											
quant	–0.063	–0.037	0.311**	0.05	0.009	−0.154*										
affect	0.024	–0.009	−0.230**	–0.062	–0.026	0.021	–0.069									
sad	0.208**	0.141*	−0.276**	−0.235**	0.042	–0.069	–0.03	0.554**								
cogproc	0.254**	0.250**	−0.151*	0.031	0.119	0.116	0.267**	–0.019	−0.215**							
insight	0.168*	0.185**	−0.220**	–0.085	–0.013	0.111	0.161*	–0.015	−0.138*	0.718**						
tentat	0.175**	0.104	–0.007	0.081	0.057	–0.096	0.226**	−0.153*	−0.171*	0.570**	0.429**					
percept	0.116	−0.230**	−0.188**	−0.264**	0.069	–0.075	0.065	0.296**	0.315**	0.095	0.095	0.232**				
see	0.140*	−0.193**	–0.069	−0.229**	–0.058	–0.062	0.074	0.237**	0.302**	0.004	–0.009	0.148*	0.799**			
feel	0.013	−0.295**	−0.138*	−0.182**	–0.031	–0.077	0.289**	0.161*	0.079	0.321**	0.257**	0.287**	0.511**	0.268**		
leisure	0.111	–0.106	−0.247**	−0.370**	–0.118	0.1	0.169*	0.185**	0.181**	0.271**	0.432**	0.137*	0.426**	0.292**	0.490**	

**Correlation is significant at the 0.01 level (2-tailed).

*Correlation is significant at the 0.05 level (2-tailed).

It should also be noted that the data volume of pre-migration was about twice as much as other datasets, We tried to cut the data volume of migration groups by half at random, and the results were the same as those reported above.

## Discussion

Using CC-LIWC, we calculated the frequencies of different psycholinguistic LIWC categories used in three groups of text by more than 200 gentry members of the Jin Dynasty. Then we run ANOVA to check whether there were significant differences between the three groups for each LIWC categories. According to the ANOVA result, the migration group is significantly different from the pre-migration group and the post-migration group in three categories of words (*tense markers, negative words, and modal particles*), which indicates the direct impact of the mass migration; the migration group is significantly different from the post-migration group in seven categories of words (*words for cognitive processes, words for insight, tentative words, words for perceptual processes, words for seeing, words for feelings, and words for leisure*), which might demonstrate the indirect impact. Combined with the psychological models of production in different words (Meyer, 1990), we’d like to discuss the psychological meaning of these words with significant differences, and verify and analyze them based on historical facts and existing relevant researches.

We found tense markers were much more frequently used in the migration group than pre-migration/post-migration group. Tense markers, related to focuspast or focuspresent or focusfuture, are deemed as “self-focused” due to their reference to the time of the user (Prior, 2003). Thereby the higher frequent use of tense markers means that they became more sensitive to tense markers and became more aware of the contrast of different times than their ancestors and descendants, showing a stronger self-focused orientation.

The Jin Dynasty is often called “a literary awareness age (Suzuki, 1989).” Apart from literature prosperity, the Western Jin is also known for its popularity of Xuanxue, which can be perceived as an ideological awareness. Such an ideological awareness may prompt the gentry at the time to take a more subjective perspective to think about writing and literature, the way things work and their position in society (Lv, 2015) and try to break away from the influences of their ancestors (Liu, 2009). The higher frequent use of tense markers in the southward gentry’s text well mirrors their self-focused way of thinking. For them, past tense markers often reminded them of the collapse of the Western Jin, so they became more aware of the present and highlighted their feelings and thoughts about the present in their text.

As negative words, they were much more frequently used in the migration group than the pre-migration and post-migration group, which indicates a self-conflicting orientation of the southward gentry (Zhao, 2021).

It is almost inevitable for the southward gentry to go through such ideological self-conflicts after fleeing to the south and seeing the homeland falling into enemy hands. It is their failed governance that led to the loss of the north. They were directly responsible for the demise of the Western Jin. Therefore, the southward gentry often used negative words to negate their pre-migration status, lives and thoughts. Their reflections on Xuanxue well embody a self-conflicting orientation. When Xuanxue prevailed during the Western Jin, most gentry members at the time advocated for governing the country by doing nothing that goes against nature. Officials like Wang (2021) Yan even took pride in not attending much to public affairs. However, after migrating to the south, many gentry members showed a totally different mentality (Fang, 2015). Such expressions represent a total repudiation of the previous reverence for Xuanxue. In fact, military expeditions launched by the Eastern Jin to recover the north can be considered as a collective manifestation of the southward gentry’s reflections on and negative attitude toward their previous governance approach of doing-nothing (Ning, 2021).

Modal particles indicate a user’s subjective opinions and feelings about a subject and can help intensify his/her attitude toward the content of a sentence or a subject (He, 2019). Such words can express not only a user’s feelings, but also subjective emotions and communicative intentions (Xie, 2015). Modal particles are much less in the migration group than in the pre-migration group and the post-migration group. This may indicates that the southward gentry’s willingness for active self-expression faded (Hu Q., 2010) after they personally experienced the Rebellion of the Eight Princes and the southward migration. They did their utmost to avoid getting themselves in trouble.

Apart from the significant differences in the use of the above three types of function words (Pennebaker, 2018), the three groups also show significant differences in the use of content words expressing psychological processes (Pennebaker, 2018). Such content words include *words for cognitive processes* (e.g., words for insight and tentative words), *words for perpetual processes* (e.g., words for seeing and for feelings), *words for emotional processes* (e.g., words for sadness), and words for personal concerns (e.g., words for leisure) (Fan, 2020). A gloomy mentality grasped the gentry, and they lost confidence in realizing the grand unification which had been normality ever since the Qin and Han dynasties (Fan, 2020). The gentry saw the northern land falling into the hands of tribal peoples, the civilization glory and political dominance they were once proud of came to an end, leading to changes to their psychological processes (Zhang, 2009). However, the southward gentry was unable to recover the lost north as they were weighed down by the fatiguing journey to the south. They tried to repress their sadness and perceptions of their miserable situation. Such psycholinguistic changes deeply impacted their descendants. This probably explains why many Eastern Jin gentry members turned to the Taoism regimen, committed themselves to explore the relationship between selfhood and nature, and began to reflect on their ancestors’ reverence for Xuanxue (Luo, 2005). Consequently, the use of words representing psychological processes such as perpetual, cognitive and emotional processes is much more frequent in their text compared with the text of their ancestors.

Such highly frequent use of words representing psychological processes is also closely related to the complicated political situation during the Eastern Jin. The Eastern Jin regime was the result of a delicate balance between the northern gentry, the southern gentry and the royal power. The three parties tried their best to avoid upsetting the balance, which weakened the southward gentry’s ambitions for grand unification. Fortunately, the southern areas provided them with great pleasure as the beautiful landscapes were totally different from the north. As a result, they turned their attention from Xuanxue to nature. While enjoying nature, they also reflected on Xuanxue and even negated human existence, which brought about the prosperity of Xuanyan poetry (poetry about Taoism and Buddhism) (Wang, 2019) and Xianyou poetry (poetry about immortals) (Mei, 1998). By immersing themselves in the creation of scenic poetries, their sadness inside was covered by the happiness on the surface (Xuemei, 2009).

This mass migration was unprecedented in terms of the number of people, the obvious family background, the long duration, and the wide area involved (Hu A. X., 2010). Therefore, its impact was bound to be huge, extensive and far-reaching. From the results of the grouping study of three groups, we creatively and quantitatively confirmed the psychological impact of the “migration” event on those who experienced the migration.

## Conclusion

It usually takes decades and even hundreds of years to shape and change the psychological state of a group of people. Natural and social factors that give rise to this change are somehow too complicated and volatile to be reproduced by any human-designed experiment (Ke, 2015). Using CC-LIWC, this paper calculated the frequencies of LIWC categories in Classical Chinese legacy text by Jin gentry members to identify the differences between different groups.

We found the direct and indirect, immediate and long-lasting psycholinguistic impacts of the mass migration on the gentry members. From the much more frequent use of tense markers, negative words and modal particles in the text of the southward gentry, we found their stronger self-focused orientation in perceptions, severer self-conflicting orientation in ideology, and abating willingness to express themselves; from the higher frequent use of words for emotional, perpetual and cognitive processes in the text of the Eastern Jin gentry, we learned why they were enthusiastic about creating Xuanyan poetry (poetry about Taoism and Buddhism), Xianyou poetry (poetry about immortals), and Shanshui poetry (poetry about landscapes). This paper shows how quantitative methods can be used to study the psycholinguistic changes of a group of people based on their text.

The innovation of this paper was that we collected a large number of self-expression texts of the gentry who related to the first mass migration of China, and We conducted psycholinguistic studies and comparisons of them. In the past, most studies on gentry were qualitative analysis from the viewpoint of selecting representatives from their writings which mostly were poems and songs. This paper started with the massive texts with more self-expression significance, such as letters, diaries, essays, etc., and quantitatively analyzes the differences in the psycholinguistic word frequency of gentry to explore their psychological changes.

Due to a lack of records, we are unable to specifically determine which self-expressive text of the southward gentry were written before or after the southward migration. Nonetheless, we adopted the closest way to make a distinction by dividing the text into three groups based on the timeline of the southward migration. And a comparative study of the three groups provided a clear insight into the psycholinguistic impacts brought by the migration. Our research shows that such quantitative methods can provide meaningful results, which provides a new way to analysis legacy text.

## Data availability statement

The original contributions presented in this study are included in the article/supplementary material, further inquiries can be directed to the corresponding authors.

## Author contributions

TZ and HL designed the research. HX conducted the research and completed the manuscript. GW, SL, and SX participated into the data analysis and manuscript revision. All authors contributed to the article and approved the submitted version.
